# Biliary Injuries Repair Using Copolymeric Scaffold: A Systematic Review and In Vivo Experimental Study

**DOI:** 10.3390/jfb16080297

**Published:** 2025-08-18

**Authors:** Salvatore Buscemi, Giulia Bonventre, Andrea Gottardo, Mariano Licciardi, Fabio Salvatore Palumbo, Giovanni Cassata, Luca Cicero, Giulia Lo Monte, Roberto Puleio, Attilio Ignazio Lo Monte

**Affiliations:** 1 Di Cristina Benfratelli Hospital, Piazza Nicola Leotta 4, 90127 Palermo, Italy; buscemi.salvatore@gmail.com; 2 Department of Precision Medicine in Medical, Surgical and Critical Care Area (Me.Pre.C.C.), University of Palermo, Via Liborio Giuffrè 5, 90127 Palermo, Italy; giulia.bonventre@unipa.it (G.B.); attilioignazio.lomonte@unipa.it (A.I.L.M.); 3 Department of Biological, Chemical and Pharmaceutical Sciences and Technologies (STEBICEF), University of Palermo, Via Archirafi 32, 90123 Palermo, Italy; mariano.licciardi@unipa.it (M.L.); fabiosalvatore.palumbo@unipa.it (F.S.P.); 4 Experimental Zooprophylactic Institute of Sicily “A. Mirri”, Via Gino Marinuzzi 3, 90129 Palermo, Italy; giovanni.cassata@izssicilia.it (G.C.); luca.cicero@izssicilia.it (L.C.); roberto.puleio@izssicilia.it (R.P.); 5 Independent Researcher, 90036 Misilmeri, Italy; giulialom@virgilio.it

**Keywords:** scaffold, biopolymers, bile duct, tissue engineering, systematic review, in vivo study

## Abstract

**Background:** Common bile duct (CBD) treatments are often associated with complications, limiting long-term efficacy. To overcome these issues, polymeric grafts have been suggested as promising alternatives, since they are highly customizable, biocompatible, and may reduce side effects frequency. **Methods:** A systematic review was conducted, interrogating MEDLINE and Cochrane Library. Next, an in vivo study involved 20 pigs, which underwent a former controlled biliary injury. To repair the defect, a *α,β-Poly(N-2-hydroxyethyl)-DL-Aspartamide* (*PHEA*)–*Polylactic-acid* (*PLA*)–*Polycaprolactone* (*PCL*) scaffold was implanted. The animals were sacrificed at one and three months for gross and histological examinations, to assess tissue integration and healing outcomes. **Results:** The systematic review highlighted that such scaffolds have shown promising results in CBD regeneration, both in single and joined applications. These findings were confirmed by the in vivo study, where the use of such scaffolds—particularly, the planar ones—led to safe and complete bile duct regeneration. Histological analysis revealed lymphomonocytic infiltrates and neovascularization, while microscopic examination showed progressive scaffold degradation accompanied by biliary tissue regeneration. **Conclusions:** Experimental results are consistent with the literature, confirming the potential of such polymeric scaffolds in aiding complete CBD regeneration and being reabsorbed shortly after. Still, further studies are needed to fully validate their translational application. **PROSPERO ID**: CRD420251115056.

## 1. Introduction

Laparoscopic cholecystectomy represents the gold standard treatment of cholelithiasis, and it is commonly preferred to the open approach because of its more favorable outcomes. However, many studies showed that this procedure is associated with an increased risk of common bile duct (CBD) injuries, with an incidence of 0.5–0.8% [1,2,3].

In CBD injury, treatment requires a multidisciplinary approach depending on the size of the lesion. Surgical options may vary from the placement of subhepatic drainage to the performance of biliodigestive anastomosis, which can restore intestinal continuity [4]. However, these interventions are associated with both short- and long-term complications [5,6,7]. Indeed, these are burdened by high morbidity and mortality rates (6.5% and 4.2%, respectively), thus reducing the patients’ Quality of Life (QoL) [8,9].

In this context, the rapidly advancing field of Tissue Engineering and Regenerative Medicine (TERM) presents potential solutions. Such an innovative field of medical sciences is constantly testing and developing new natural or synthetic 3D biocompatible and resorbable scaffolds that may support and promote CBD regeneration; indeed, these can be fine-tuned to properly fit a certain microenvironment [10] and its tissue characteristics [11]. Doing so, such scaffolds may provide support for adhesion and implantation of various cellular populations, promoting the growth of new tissues with the same morpho-functional features as the native ones [12].

Moreover, these scaffolds are designed to provide always just the right grade of mechanical support; in the early phases of regeneration, they maintain their mechanical integrity to provide strong support to the growing tissues against the forces acting in vivo; eventually, they are completely degraded, allowing for tissue remodeling [10].

Still, previous studies underlined that natural and autologous grafts, such as those derived from animal intestines, are plagued by early degradation and immune rejection, hampering long-lasting performance [13,14]. Therefore, new polymeric scaffolds are in development. Among these, the copolymeric scaffold made by *α,β-Poly(N-2-hydroxyethyl)-DL-Aspartamide* (*PHEA*), *Polylactic acid* (*PLA*) and *Polycaprolactone* (*PCL*)—namely, PHEA-PLA-PCL, is emerging as a valuable alternative, designed to ensure biocompatibility and long-term stability. Indeed, several studies previously carried out by the authors proved that PHEA-PLA-PCL has a high mechanical resistance and good biocompatibility. Such assessments regarded bile duct regeneration in the rabbit model and arteriovenous fistula in the pig model [15,16,17].

To properly evaluate PHEA-PLA-PCL scaffold characteristics in the context of CBD reconstruction, a systematic review of the literature will be conducted, thus gathering and analyzing existing evidence on its behalf and, therefore, providing a comprehensive overview of the subject. Moreover, this will be compared to the experimental results obtained in a proper in vivo study on pigs; here, the ability to withstand the lytic action of bile and to result in the complete CBD regeneration will be assessed. The goal will be to identify best practices, potential issues, and optimization criteria to allow the application of such scaffolds in human biliary diseases.

## 2. Results

### 2.1. Systematic Review

Table 1 depicts the results of the systematic review, along with the Quality Assessment (QA) of each inserted article. To note, QA results were low, as the systematic review only included D5 articles, i.e., reviews and preclinical studies. Nonetheless, the reported information can still be useful for making initial assessments about the experimental efficacy of such devices.

#### 2.1.1. Analysis of In Vitro Studies

Several in vitro studies assessed the effectiveness of polymeric scaffolds for CBD tissue engineering. Xu [21] investigated a PLGA–BaSO_4_ scaffold, noting that its structural integrity remained stable during the first two weeks of bile exposure, followed by a predictable degradation pattern via hydrolysis, without toxic byproducts. This was key in suggesting its potential for safe in vivo translation. Zhou [23] evaluated electrospun PLGA scaffolds seeded with CBD-derived endothelial cells from swine BMCs, showing high cellular viability, uniform attachment, and biliary epithelial marker expression, without evidence of cytotoxicity. The scaffold maintained its fibrous architecture for up to two weeks, making it suitable for bile duct support. Kim [25] applied electrospray deposition to create sorafenib-loaded PCL films on metallic stents, aimed at delivering a localized chemotherapeutic effect against cholangiocarcinoma. In vitro tests showed a sustained drug release for up to 30 days, significantly reducing cancer cell proliferation and angiogenesis, and suggesting its utility for malignant biliary obstruction. Cai [26] developed PLA/PCL drug-eluting stents embedded with EDTA and SC to promote CBD stone dissolution. In ex vivo perfusion models mimicking bile flow, 50% EDTA/SC stents achieved up to 26.2% stone mass loss over ~13 weeks, balancing degradation and therapeutic efficacy. Notably, higher concentrations accelerated degradation but eventually reduced overall effectiveness, underlining the importance of polymer tuning. Huang [28] expanded upon such a scaffold by comparing three fabrication techniques (i.e., dip coating, coaxial electrospinning, and their combination), revealing that the combined method ensured the most controlled and prolonged drug release, resulting in superior stone dissolution in vitro. Buisson [32] employed a multilayered 3D-printed PCL scaffold with distinct fibrous and microporous regions, seeded with hCdH-Chols. The inner fibrous layer promoted differentiation and epithelialization within 14 days, while the outer microporous layer provided mechanical robustness. Finally, Hallett [33] validated the functionality of a novel human biliary epithelial cell (hBEC) line derived from cadaveric liver tissue, demonstrating high proliferative and clonogenic capacities. When seeded on electrospun PCL scaffolds, hBECs retained their cholangiocyte phenotype, further confirming the biocompatibility and regenerative suitability of these polymeric constructs.

#### 2.1.2. Analysis of In Vivo Studies

A substantial body of research supports the safety and functionality of scaffold-based CBD substitutes in animal models, confirming the biocompatibility, mechanical resilience, and regenerative potential of such functionalized devices. Miyazawa [18] reported that PCL–PLA/PGA scaffolds, whether seeded with BMCs or not, supported full epithelialization and structural regeneration in pigs after six months, with no adverse events (AEs) or histological discrepancies between groups. Laukkarinen [19] introduced a radiopaque, biodegradable PLA–BaSO_4_ stent in a porcine model, significantly reducing bile leakage and eliminating the need for secondary endoscopic removal. The stents degraded fully by six months, and histological analysis revealed only mild inflammation, demonstrating both functional and safety advantages over non-biodegradable PE controls. Xu [21] found that radiopaque PLGA scaffolds implanted in dogs maintained bile duct patency, induced minimal inflammation, and allowed normalization of hepatic biomarkers by week five, confirming their degradability and biocompatibility in a large animal model. Aikawa [22] showed that even unseeded PCL–PLA/PGA patches could regenerate ductal tissue in pigs, with cuboidal columnar epithelium formation and no residual fibrosis or scarring after four months. Kim [25] validated the in vivo anti-tumor efficacy of sorafenib-loaded PCL stents in murine xenograft models, with marked tumor inhibition and apoptosis, without systemic toxicity or structural failure upon stent deployment. Buscemi [16] reported that PHEA-PLA/PCL scaffolds resisted bile corrosion while facilitating epithelial and glandular cell regeneration in rabbits, indicating strong regenerative performance even in smaller animal models. Huang [28] confirmed just the biocompatibility of EDTA/SC-eluting stents in vivo, not being able to test stone mass loss due to a high mortality shown in a preliminary test, when scaffolds were placed with biliary stones. Girard [29] designed a radiopaque, resorbable biliary stent composed of PLA/PEG with TIB–PCL. In vivo and in vitro studies obtained similar results, with a quasi-linear degradation of the PLA/PEG component, only late structural fragmentation, a fibrous encapsulation by 6 months, and preservation of mechanical properties for 4 weeks, thus ensuring duct patency during healing. Cadaveric testing by four surgeons yielded a 75% implantation success rate, confirmed by CT imaging. Buisson [32] demonstrated significantly higher survival and functional recovery in rabbits implanted with hCdH-Chol-seeded PCL scaffolds compared to unseeded controls, which all died within five days. Histological analyses confirmed integration and absence of stricture formation. Kim [34] divided 14 pigs into a 3D-printed PCL/BaSO_4_ stent group and a sham group; while radiological patency was confirmed, complications included three early deaths (one in the test and two in the control group), stent migration, fracture, and moderate inflammation. Finally, Valderrama-Treviño [37] demonstrated long-term scaffold integration over an 18-month period using PLGA–PCL–Gel CBD scaffold in pigs, with full epithelial regeneration and sustained bile duct functionality, supporting the potential of prolonged regenerative applications.

#### 2.1.3. Analysis of Review Articles

The included reviews offered comprehensive overviews of scaffold technologies, biomaterials, and tissue engineering approaches for bile duct regeneration and disease modeling. Aikawa [20] and Justin [27] underscored the regenerative capacity of bioabsorbable polymer scaffolds, particularly PCL–PLA/PGA composites, in promoting early tissue remodeling and epithelial recovery, while also discussing the importance of novel surgical techniques and cell-based therapies. Kasuya [24] compared collagen and PLGA-based matrices for bile duct formation in vitro, showing that although collagen supported functionality, it lacked integration with other hepatic structures—an issue resolved by PLGA membranes, which demonstrated improved modularity and compatibility for 3D co-culture systems. Brevini [30] reviewed the potential of synthetic materials like PLA in constructing scaffold-based bile ducts, highlighting their adaptability and mechanical control, yet pointing out the limited number of preclinical validations available to date. Wang [31] provided a detailed evaluation of TEBDs, emphasizing the necessity for scaffolds that balance biocompatibility with mechanical strength and tunable degradation rates. The review eventually advocated hybrid scaffolds, i.e., the ones that combine natural and synthetic elements, as promising tools to mimic physiological bile duct architecture. Miyazawa [35] proposed a classification of bile duct substitutes based on their source materials and underscored critical design factors such as wound healing dynamics, biliary fluid properties, and tissue integration at the anastomotic site—all essential for ensuring scaffold functionality and long-term efficacy. Finally, De Siervi [36] focused on the utility of synthetic 3D scaffolds in modeling primary liver cancer, arguing that their mechanical reproducibility and controlled degradation outperform natural matrices in experimental standardization and therapeutic testing, further confirming the translational potential of these bioengineered platforms.

### 2.2. In Vivo Study

All the animals completed the planned follow-up without spontaneous deaths or the need for early euthanasia due to clinical deterioration. No fatal AEs occurred during the observation period.

As yet described in the method section, the 20 animals were divided as reported in the following Table 2.

#### 2.2.1. Obtained Results

Hereafter, the results obtained in the in vivo study, divided into groups:

- Group A: Gallbladder wall planar scaffold

All pigs survived the procedure, and no complications occurred. Animals were sacrificed after three months, and a cholecystectomy was performed. The structure of the scaffold was macroscopically indistinguishable from the gallbladder.

**Figure 1:**
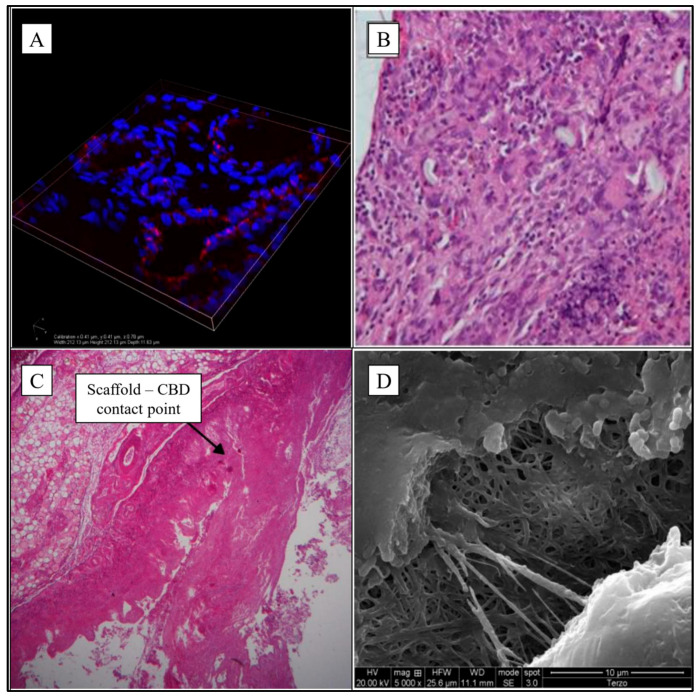
Microphotographs of implanted scaffolds. (**A**) 3D organization of endothelial cells with confocal microscope (*Nikon a1*—width 212.13 µm, height 212.13 µm, depth 11.63 µm—cellular nucleus in blue, antibodies anti-CD31 in red); (**B**) Gallbladder-jejunal scaffold histological examination (EE20×) shows intense chronic inflammatory infiltration in the portion of the scaffold; (**C**) CBD tubular scaffold histological examination shows the contact point of proximal anastomosis (EE5×); (**D**) the scaffold patch of CBD is partially degraded at electron microscopy 5000×. Sources: (**A**,**C**,**D**) are reprinted from Ref. [38]; (**B**) = owned by the co-authors.

Histological examination showed the complete reconstitution of the gallbladder mucosa with a simple columnar epithelium and a widespread monocyte infiltration. Neoangiogenesis was evidenced, as well as an intense inflammatory response. The scaffold sample was examined by immunofluorescence using anti-CD31 monoclonal antibodies. Staining revealed the presence of numerous vessels around the fibrous tissue. Serial imaging along the z axis allowed us to determine the correct organization of the vessels and confirmed the good response in terms of neoangiogenesis (Figure 1A).

- Group B: Gallbladder-jejunal tubular scaffold

The postoperative course was regular, and sacrifice of animals was scheduled at three months.

An intense phlogistic reaction was found in the new bile duct made up by the scaffold, which was only partially reabsorbed. There was evidence of a foreign body granulomatous reaction, confirmed by the histological examination that showed an intense chronic inflammatory infiltration in the portion of the scaffold (lymphocytes, macrophages and giant cells) (Figure 1B).

- Group C: CBD tubular scaffold

The postoperative course was normal. Laparotomy was performed after three months, and the scaffold and its anastomoses were in place without leaking. The microscopical exam of the scaffold revealed signs of integration between the scaffold and CBD (Figure 1C).

- Group D: CBD patch

Given that the CBD lesion to be repaired in this group was planar and only 2 cm in size, it was decided to sacrifice the animals just one month after surgery, in order to evaluate the early effects of the implant. Although there was macroscopic evidence of the complete integrity of the CBD without any scaffold adhesion left, the patch was only partially degraded. SEM examination further confirmed this, showing an almost intact fibrillar structure after only one month (Figure 1D).

#### 2.2.2. Semiquantitative Histopathological Analysis

Semiquantitative histopathological analysis confirmed the microscopic observations, allowing for an objective comparison of the regenerative process obtained in the different experimental groups. Table 3 shows a semiquantitative comparison of the results.

As could be seen, groups A (planar on the gallbladder wall) and D (CBD patch) showed the best results in terms of complete epithelial regeneration and intense neovascularization, accompanied by just mild or moderate inflammation, compatible with a physiological response to the biomaterial. These data suggest that in limited parietal defects, the regenerative process is faster and more favorable, probably due to the presence of vital tissue margins that facilitate cell colonization and vascularization.

In contrast, in groups B and C, where the defect was reconstructed with complete tubular scaffolds, a more intense inflammatory reaction was observed, associated with partial epithelial regeneration and less advanced scaffold degradation. This could be attributed to the greater structural complexity of tubular regeneration, which requires more extensive neoangiogenesis and centripetal cell infiltration, conditions that are more difficult to achieve in a three-dimensional implant without residual epithelial support.

Focusing on scaffold degradation, the results show that planar scaffolds degrade faster than tubular ones, thus providing structural support to the regenerating tissue in the early stages of the healing process and beginning to degrade from the third month onwards. On the contrary, after three months, tubular scaffolds are still only partially degraded, remaining in the tissues for a longer period.

These results seem to suggest that the use of PHEA-PLA+PCL is more effective in partial bile duct defects, while in complete reconstructions, prolonged follow-up or modification of the material formulation may be necessary to optimize scaffold degradation and tissue response.

## 3. Discussion and Conclusions

TERM technologies rely on biomimetic and biocompatible materials to create artificial substitutes, aimed at restoring and maintaining the normal function of ill and damaged tissues or organs without any AEs. Particularly, the use of synthetic polymers has several advantages, primarily relative to their fine-tuning capabilities. By, for instance, the formation of copolymers, it is possible to modulate their chemical, physical and mechanical properties. Even their porosity may be regulated to resemble one of the natural tissues. Finally, the presence of free chemical groups can be used to incorporate other substances (e.g., heparin, antibiotics and growth factors) by direct binding [39].

Previous studies proved the ineffectiveness of natural grafts in CBD application, due to frequent AEs and lower tuning capabilities. Indeed, CBD reconstruction needs to be pursued carefully to avoid medium and long-term serious complications, such as biliary fistulas and stenosis with obstructive jaundice [40]. Therefore, polymeric scaffolds—although not yet utilized in clinical practice but just assessed in preclinical studies—may be the ones of choice in this context. This is exactly the hypothesis this paper aimed to confirm, through a systematic review and an in vivo study.

The systematic review confirmed the growing interest in synthetic polymers—particularly PCL, PLA, PGA and their copolymers—for the engineering of bile duct substitutes. In vitro results highlighted the favorable mechanical behavior, degradation profiles, and cytocompatibility of such materials, especially when combined with functional molecules or layered architectures that promote cellular viability, tissue-specific differentiation and the treatment of the underlying pathology. In vivo studies consistently demonstrated good biocompatibility and successful regeneration of biliary structures across several animal models, showing restored bile flow, normalized liver function markers, and histological reconstitution of ductal epithelia. However, clinical translation is still unachieved, as no clinical studies were retrieved. Consequently, the overall quality of evidence is low.

Notably, some studies did report relevant complications, such as post-operative mortality, inflammation, stent migration, and mechanical failure. Yet these AEs were not consistently linked to a specific polymer or strategy. In fact, some techniques that yielded poor outcomes in certain studies—e.g., drug-eluting stents or unseeded scaffolds—proved effective in others. This heterogeneity in protocols, models, and evaluation endpoints hinders the possibility of establishing which combination of material, structure, and cell seeding approach is most promising. To this end, comparative studies under controlled and standardized conditions are urgently needed, particularly in large animals and in long-term follow-up settings, to define clear efficacy and safety profiles for each strategy. To support the design of such future studies, researchers may benefit from the methodological recommendations outlined in the included reviews; for instance, the reviews by Justin [27] and Miyazawa [35] offer structured frameworks for scaffold development with therapeutic intent, while those by Wang [31] and De Siervi [36] are more suitable for disease modeling applications, particularly in the context of cancer research and drug testing.

The results of the preclinical study show that the PHEA-PLA/PCL scaffold can stimulate the regeneration of portions of pig biliary tissue. Moreover, microscopic analysis at three months showed a progressive degradation of the scaffold and a concomitant regeneration of the biliary tissue, as shown by the presence of new biliary epithelium in the implantation site under optic microscopy. Therefore, the material proved to be resistant to the lytic action of bile, as well as conducive to tissue regeneration. This might initially suggest possible clinical use of such a polymer conduit to achieve CBD regeneration in human patients. However, long-term exposure to bile acids in humans, where biliary pH and microbial flora may differ significantly, remains untested. Dedicated durability studies will be needed to confirm the scaffold’s chemical and mechanical stability in human settings.

In detail, in all animals, there was no obliteration of the new ducts, and anastomoses between the tubular scaffold and the CBD were successful from a technical point of view. Nonetheless, microscopic examination of the surgical specimens did not show complete integration between the native tissue and the scaffold, probably due to the short follow-up. Therefore, three months does not seem to be enough for the complete degradation of the scaffold and its replacement with a new duct. Still, no sign of leakage or stenosis was noted, and there was partial integration in some regions, indicating the initial stimulation of the regenerative process.

The functional properties of the scaffold, its structural resistance to bile, and its ability to induce tissue regeneration are further supported by the results from the CBD patch model. In this group, the limited size of the defect likely favored a more rapid and complete regeneration, with full-depth tissue reconstitution and scaffold resorption already achieved after one month. This suggests that the scaffold is more effective in the treatment of partial defects, where native tissue can support colonization and healing.

This interpretation is corroborated by the semiquantitative histopathological analysis, which allowed direct comparison of the regenerative processes among the four experimental groups. The planar scaffold (group A) and the patch model (group D) yielded the best results, with intense neovascularization, complete epithelial regeneration, and only mild to moderate inflammation—responses that are consistent with physiological healing. Conversely, the groups treated with complete tubular scaffolds (groups B and C) exhibited more intense inflammatory infiltrates, delayed scaffold degradation, and incomplete epithelial repair. This outcome likely reflects the greater complexity of tubular regeneration, which lacks residual epithelial support and depends on more demanding centripetal infiltration and vascularization processes. In this context, the scaffold degradation may trigger an exaggerated inflammatory response that could potentially slow the healing process. Future studies shall evaluate this phenomenon in greater detail to avoid chronic inflammation or fibrosis.

Such modulation could be achieved by refining scaffold features such as wall thickness, fiber alignment, porosity, and polymer composition, to facilitate better vascularization and centripetal colonization of the lumen. Moreover, improvements in surgical technique, such as minimizing manipulation, ensuring tension-free anastomosis, and standardizing suture type, may also influence outcomes and graft integration.

Although the preliminary results give reason to be cautiously optimistic, further technical adjustments are needed to assess the ability of the polymeric conduit to guide the complex regeneration process of tubular structures such as the CBD. On the other hand, the planar and patch versions of the same material have shown encouraging performance in regenerating damaged biliary tissue. Still, independently of its geometry, the material showed resistance to the lytic action of bile, and its 3D structure allowed performing complex anastomosis, with calibers as small as 4 mm. Indeed, from a surgical standpoint, the material was easy to handle, resisted suture, and showed favorable elasticity and porosity. Moreover, the scaffold demonstrated excellent biocompatibility, with biodegradation mediated by a moderate inflammatory response, which in turn appeared to support tissue regeneration through vessel ingrowth and immune cell recruitment. Particularly, no AEs related to residual scaffold fragments were observed during follow-up, although this was limited to a maximum of three months; longer studies are required to rule out late complications such as foreign body granulomas or persistent inflammation.

Still, it is particularly relevant that such results were achieved in the biliary environment, where regenerative stimuli are intrinsically poor, given the absence of circulating progenitor cells or reparative elements. Therefore, the scaffold’s ability to promote neovascularization and epithelialization without anatomical or functional complications suggests its suitability for further development.

This study has several limitations: most included studies were of low quality, often lacking standardized endpoints or controls, which precluded meta-analysis. The pig model, while anatomically relevant, differs from humans both at the cytological and physiological levels, and only a small sample of healthy pigs was tested for a limited follow-up, thus, limiting the strength of the conclusions. Finally, no molecular markers of regeneration were evaluated, nor was immune cell infiltration assessed beyond standard histology.

Nonetheless, this work provided a comprehensive literature overview and an original observational study that may contribute to advancing the current knowledge in the field. As such, it may stimulate further investigations into the cellular and molecular mechanisms underlying bile duct regeneration. A deeper understanding of these dynamics could enable the identification of reliable biomarkers, allowing for more precise monitoring and modulation of the regenerative process.

In conclusion, this study demonstrated that PHEA-PLA+PCL scaffolds—and, more broadly, synthetic polymer-based materials—exhibit favorable properties for supporting biliary tissue repair. Given their biocompatibility, mechanical resilience, and degradation profile, as well as the scalability of their manufacturing processes, these biomaterials may represent promising candidates for future clinical applications in hepatobiliary surgery. Nonetheless, further research is mandatory before clinical implementation, with particular attention to long-term safety, immunological and molecular response, and effectiveness in diseased biliary environments. Particularly, such research should aim at directly comparing the various polymers and scaffold architectures tested to date, under standardized conditions and with long-term follow-up, to identify the most suitable formulations and functionalizations (e.g., with cellular components or growth factors) for clinical translation.

## 4. Materials and Methods

### 4.1. Systematic Review

#### 4.1.1. PRISMA 2020 Flow Diagram and Checklist

**Figure 2:**
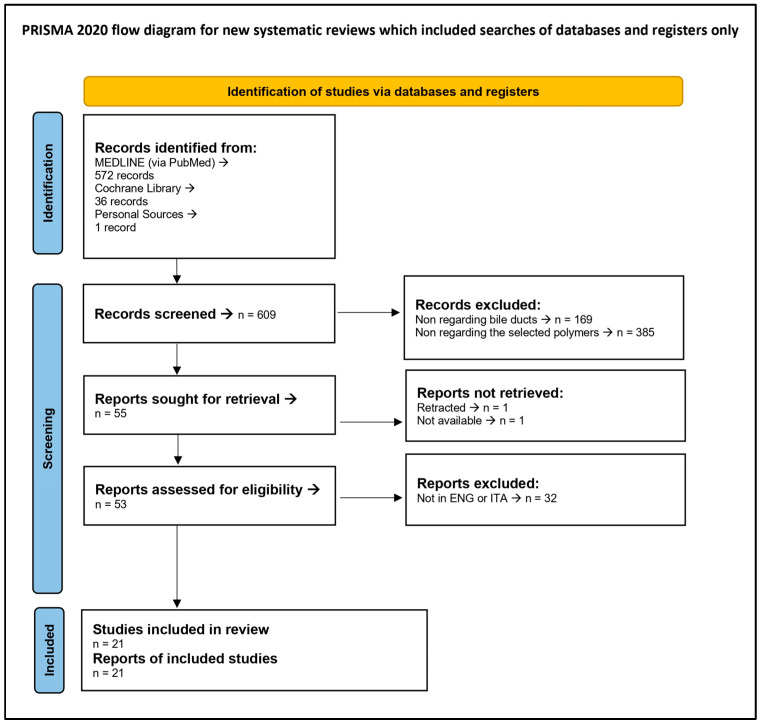
PRISMA 2020 Flow Diagram.

Figure 2 presents the PRISMA 2020 Flow Diagram, while the PRISMA 2020 Checklist is reported in Table S1.

#### 4.1.2. Research Strings

All research was conducted on 18 March 2025, using both *MEDLINE* (via *PubMed*) and *Cochrane Library*.

- *PubMed* String → ((“Bile Ducts”[Mesh] OR “Biliary Tract”[Mesh] OR “Common Bile Duct” OR “CBD” OR “CBD injuries” OR “CBD repair” OR “Biliary injury repair” OR “Bile duct regeneration” OR “Biliary reconstruction”) AND (“Polymeric scaffold” OR “Bioabsorbable polymer” OR “Biodegradable scaffold” OR “Polycaprolactone” OR “PCL” OR “Polyhydroxyethyl aspartamide” OR “PHEA” OR “Polylactic Acid” OR “PLA” OR “Polylactide” OR “Tissue Engineering” OR “Regenerative Medicine”)).
  Used filters → Published in the last 20 years.
  Number of Results → 572.
- *Cochrane Library* String → (See Table 4).
  Used filters → Published in the last 20 years.
  Number of Results → 36.

#### 4.1.3. Screening

Once the 608 results were obtained, they were manually and independently screened by two authors (AG and GB) and their work was further evaluated by a third author (AILM). The screening was conducted according to the following inclusion criteria:

- Only papers in ENG or ITA.
- Only regarding bile ducts.
- Only regarding at least one of the following polymers:PLA, PCL, PHEA.

Doing so, 20 articles were included, along with one additional relevant paper [22], identified through personal knowledge and expertise.

#### 4.1.4. Data Extraction

Data extraction was manually conducted by one author (AG) and his work was further evaluated by two other authors (GB and AILM). It covered the following parameters:

- First Author
- Publication Year
- PMID or DOI
- Type of Study
- Technical Details
- Results

#### 4.1.5. QA and Data Synthesis

QA of included articles was manually and independently conducted by two authors (AG and GB) and further evaluated by a third author (AILM). Particularly, since both reviews and in vivo studies are included, only the *modified presentation of the Oxford Centre for Evidence-Based Medicine* (*OCEBM*) levels of evidence scale [41] is adopted for any article. In detail, it rates the type of study by assessing the grade of recommendation (with A representing the maximum grade and D the minimum) and level of evidence (with 1 representing the maximum level and 5 the minimum). In this way, for each type of study, an ascending alphanumeric value will be produced, inversely proportional to the quality of the article in question. Therefore, the highest quality will be “Grade A—Level 1” (shown as “A1”) studies, while the lowest will be “Grade D—Level 5” (i.e., “D5”) studies.

Then, to further evaluate in vivo controlled studies, the *Systematic Review Centre for Laboratory animal Experimentation* (*SYRCLE*) risk of bias tool [42] is adopted, giving 0 point for “no”, 1 point for “unclear” and 2 points for “yes. By applying this tool, a scoring between 0 and 20 (reported as “value/20 points”) will be obtained, which is directly proportional to the quality of the work: the higher the quality, the higher the score obtained.

Finally, data synthesis was performed via *ChatGPT* (vers. *4* & *4o*) by AGo. The obtained drafts were checked and re-elaborated by the same author. The final synthesis was checked by GB and AILM, then reported in Table 1.

### 4.2. In Vivo Study

#### 4.2.1. Scaffolds Preparation

Polymeric scaffolds were prepared in the *Biocompatible Polymers Laboratory* of the Biological, Chemical and Pharmaceutical Sciences and Technologies Department (STEBICEF) of the University of Palermo. The starting polymer was PHEA, a biocompatible synthetic polymer already used as a drug carrier [43]. This polymer was combined with PCL and PLA to obtain the final scaffold by electrospinning [44]. As previously described [16,17], a 50:50 *w*/*w* mixture of PHEA-PLA and PCL was subjected to electrospinning at a voltage of 6–15 kV, a speed of 0.8–1 mL/min, and an extrusion distance between the needle and collector of 15–20 cm. The resulting nanofibers (500–1000 nm) were assembled to form tubular structures with a wall thickness of approximately 0.6 mm. At the end of the manufacturing process and before use, the scaffolds were washed repeatedly, vacuum dried, and sterilized. This procedure has already been shown, both in vitro and in mouse models, to produce biocompatible, structurally stable scaffolds capable of stimulating the growth of epithelial and subepithelial layers of the bile ducts. For this reason, this study aimed to test this protocol on a porcine model, in order to have a study model as similar as possible to humans, with a view to subsequent translation to actual clinical studies.

#### 4.2.2. ARRIVE Checklist, Ethics Statements and Project Rationale

In Table S2, the *Animal Research: Reporting of In Vivo Experiments* (*ARRIVE*) *Checklist* is reported, fulfilled as needed. Furthermore, all animal experiments followed the *ARRIVE Guidelines*.

Study design was submitted to the Animal Welfare Body (O.P.B.A.) of the “A. Mirri” Institute for approval (according to Italian Legislative Decree No. 26 of 4 March 2014 transposing Directive 2010/63/EU on the protection of animals used for scientific purposes).

The “3R Principles” were used as a rationale for developing this study:

- Replacement → The pig model was selected for its high anatomical and physiological homology with humans, making it the most appropriate for testing bile duct regeneration using synthetic scaffolds. There are no validated in vitro or ex vivo alternative methods capable of comprehensively simulating the required tissue, immune, and biomechanical responses.
- Reduction → Although this is an exploratory study, the use of the minimum number of animals necessary to obtain meaningful data was ensured, in line with what is reported in the literature for similar models. The research team’s previous experience and a critical review of the literature allowed the experimental design to be optimized, reducing the need for unnecessary replications.
- Refinement → All surgical procedures were performed under general anesthesia, with subsequent postoperative pain management by the veterinarian, who applied an updated analgesic protocol and humane endpoint criteria. This minimized animal suffering and stress, ensuring compliance with the latest ethical standards.

This approach ensured maximum ethical and methodological compliance in the use of laboratory animals, promoting the reliability and reproducibility of the experimental results.

#### 4.2.3. Study Subjects and Rationale for Surgery and Follow-Up

Study subjects were 20 healthy Mini-Pigs, all females of about 20 kg of weight and 4 months of age, not previously treated. No randomization, blinding, control for confounders, nor *a priori* sample size calculation were performed. Models were divided into four groups of five subjects each to test different reconstruction techniques. Therefore, they underwent a surgical bile duct injury, which was immediately repaired with interposition of a tubular or planar PHEA-PLA+PLC scaffold. Both 2 cm^2^ planar scaffolds and 4 mm × 40 mm tubular scaffolds are used. In both scaffolds, the wall thickness was 0.6 mm. All scaffolds obtained were washed several times with bidistilled water, then dried under vacuum and sterilized.

All surgical procedures were carried out on animals under adequate general anesthesia [45]. After surgery, all animals received antibiotic treatment for 3 days and were housed at the “A. Mirri” Experimental Zooprophylactic Institute of Sicily, where the personnel monitored each animal’s stay, taking care of its feeding and ensuring optimal recovery from surgery. The sacrifice of the animals was scheduled for one or three months, depending on the surgical procedure, except in case of worsening of clinical conditions.

Histological examination of the grafted sections was performed to assess the host’s response and the degree of tissue regeneration. In Group A, immunohistochemical examination was performed to search for specific endothelial markers by using Ig anti-CD31.

#### 4.2.4. Details of Experimental Groups

**Figure 3:**
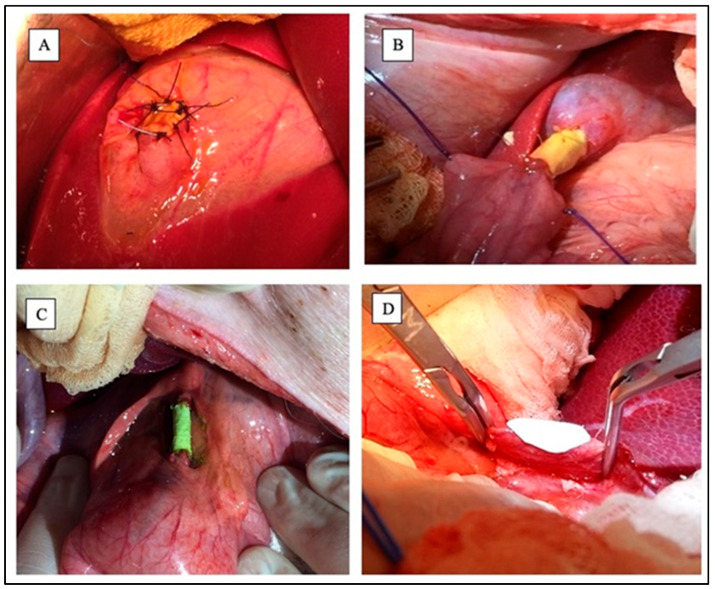
Experimental groups during surgeries. (**A**) Gallbladder wall planar scaffold; (**B**) Gallbladder-jejunal scaffold; (**C**) Common Bile Duct tubular scaffold; (**D**) Common Bile Duct patch during suturing. Sources: (**A**–**C**) are reprinted from Ref. [38]; (**D**) = owned by the co-authors.

Experimental groups were as follows:

- Group A: Gallbladder wall planar scaffold
  The fundus of the gallbladder was clamped, then an incision of about 2 cm^2^ was made. PHEA-PLA+PCL planar scaffold was sutured to replace the portion of the gallbladder wall by means of interrupted 5-0 absorbing monofilament stitches (Figure 3A).
- Group B: Gallbladder-jejunal tubular scaffold
  The gallbladder was isolated and a jejunal loop was mobilized. Both the gallbladder-scaffold and the scaffold-jejunal anastomoses were obtained by continuous suture with absorbing monofilament (Figure 3B).
- Group C: CBD tubular scaffold
  A 3 cm length tract of the distal part of the CBD was replaced with a tubular scaffold. Both proximal and distal anastomoses were obtained by continuous suture using absorbing monofilament (Figure 3C).
- Group D: CBD patch
  A wedge choledochotomy was performed. The scaffold was then placed and sutured with absorbing monofilament stitches to replace the CBD wall (Figure 3D).

## Figures and Tables

**Table 1:** Table of Results.

First Author (Year) [Ref]	Type of Study ( QA )	Technical Details	Results
**Miyazawa (2005) **[18]	In vivo (D5; 13/20 points)	Artificial bile duct reconstruction in pigs, using a Bile Duct Organoid Unit (BDOU) by seeding a bioabsorbable polymer (made with PCL–PLA copolymer reinforced with Polyglycolic Acid (PGA) fibers) and seeded with Bone Marrow Cells (BMCs).	All 18 pigs implanted with BDOU (both seeded and unseeded with BMCs) survived without complications until sacrifice. Histological analysis showed complete epithelialization and structural resemblance to native bile ducts by 6 months, indicating successful integration of the implants. No significant differences were noted between seeded and unseeded groups.
**Laukkarinen (2007) **[19]	In vivo (D5; 12/20 points)	A self-expanding, radiopaque, biodegradable biliary stent composed of PLA blended with 23% barium sulphate (BaSO_4_) was tested in a porcine model. The stents were produced via melt spinning and braided into a tubular mesh with 6–7 mm diameter and 50 mm length. Six pigs received PLA-BaSO_4_ stents endoscopically after cholecystectomy-induced cystic duct leakage; six control animals received polyethylene (PE) stents.	One control animal died from biliary peritonitis. The biodegradable stent group showed significantly reduced total bile leakage and earlier drain removal compared to controls. By 6 months, all PLA stents had degraded and were no longer detectable. Conversely, one out of five PE stents was still in place at 6 months; it was clotted and dilatated. Histological analysis revealed mild inflammatory reaction in both groups, with no significant differences in bile duct structure or function, nor foreign body reaction or obstruction. The PLA stent was considered safe, effective and advantageous, since it eliminates the need for a second endoscopy.
**Aikawa (2007) **[20]	Review (D5)	Extrahepatic bile duct regeneration; assessment of bioabsorbable polymer (made with PCL-PLA copolymer reinforced with PGA fibers) seeded with BMCs, along with novel therapies for biliary diseases.	Bioabsorbable polymer tubes seeded with BMCs facilitated the regeneration of the bile duct in the extrahepatic model, forming a structure like the native one six months post–implantation. In discussions, the authors made assessments regarding bioabsorbable biliary stent, novel surgical procedures and cell therapies.
**Xu (2009) **[21]	In vitro and in vivo (D5)	A biodegradable tubular scaffold composed of poly(lactic-co-glycolic acid) (PLGA), functionalized with barium sulfate (BaSO_4_) for radiopacity. The scaffold measured 10 mm in outer diameter and 30 mm in length. In vitro degradation was assessed by immersion in fresh human bile, while in vivo performance was evaluated through implantation in six canine models.	In vitro, the scaffold showed stable mechanical properties for 2 weeks, followed by accelerated degradation over the next 4 weeks. PLGA degraded from the luminal surface outward via hydrolysis, releasing lactic acid (LA) and glycolic acid (GA), which may be easily removed from the body by normal metabolic pathways. In vivo, no bile leakage or migration was observed. Radiopacity disappeared by week 5, indicating complete degradation and bile flow restoration. Blood tests normalized by week 5. Histology revealed early granulation and epithelial hyperplasia in week 1, mild chronic inflammation and bile encrustation in week 4, and mucosal repair with minimal residual inflammation by week 8.
**Aikawa (2010) **[22]	In vivo (D5)	Bile duct regeneration after injury; unseeded PCL–PLA copolymer reinforced with PGA fibers.	All recipient pigs survived the duration of the experiment. Initial observations post-surgery showed no signs of jaundice or complications. After five weeks, the patches were not visually identifiable; histological analysis at this level revealed inflammatory cell infiltration, fibrous connective tissue with early development of glandular structures and no epithelial regeneration. By four months, the graft sites demonstrated a well-developed cuboidal columnar epithelium formation, resembling native bile duct tissue; this indicated successful epithelial and structural regeneration, even with a subepithelial layer richer in connective tissue.
**Zhou (2013) **[23]	In vitro (D5)	An electrospun PLGA scaffold was evaluated for its compatibility with CBD endothelial cells derived from swine BMCs. The seeding was pursued to assess cell viability, morphology, adhesion, and phenotype over a 7-day culture period.	Cells adhered well to the PLGA scaffold, maintained normal morphology, and expressed biliary epithelial markers. SEM showed maintained structural integrity up to 2 weeks, and both uniform cell attachment and proliferation across the scaffold surface. No cytotoxic effects were observed, and the PLGA matrix supported endothelial differentiation and viability, confirming its potential as a biomaterial for CBD tissue engineering applications.
**Kasuya (2012) **[24]	Review (D5)	Liver reconstruction in vitro; to form bile ducts, two strategies are tested: Biliary Epithelial Cells (BECs) were seeded between two layers of collagen gel, or onto Poly(D,L-Lactide-co-Glycolide) (PLGA) microporous membranes.	The results indicated successful formation of bile ducts with the collagen matrix approach, showing typical cellular markers and functionality, but the thick gel layers prevented co-culturing with other hepatic-like structures. The PLGA membrane approach overcomes this issue, showing enhanced integration with other gel layers containing hepatic-like structures. Moreover, several parameters of such polymeric membranes allowed fine-tuning of the final 3D liver tissue model.
**Kim (2013) **[25]	In vitro and in vivo (D5; 10/20 points)	Testing of chemotherapeutic (CT) drug-eluting biliary stent, fabricated by electrospray deposition of sorafenib-loaded PCL films onto a metal mesh. Anticancer activity was evaluated in vitro on human cholangiocarcinoma cells (HuCC-T1) and in vivo in a mouse xenograft model.	The sorafenib-loaded PCL stents showed sustained drug release for up to 30 days, with dose-dependent inhibition of cancer cell proliferation, migration, invasion, and angiogenesis. In vivo, the implants suppressed tumor growth and induced apoptosis without significant toxicity. Finally, the CT-loaded PCL matrix maintained structural integrity during stent deployment test with endoscopic equipment.
**Cai (2014) **[26]	In vitro and ex vivo (D5)	Development of drug-eluting stent composed of plastic coated with PLA and PCL; these polymers are embedded with EDTA and sodium cholate (SC), to promote biliary stone dissolution. The study used an ex vivo perfusion model simulating human bile flow, employing porcine bile and human CBD stones.	Dose-dependent stone dissolution is found. The 50% EDTA/cholate stents achieved the highest stone weight loss (26.2%), balancing effective dissolution with optimal membrane degradation time (~13 weeks). Higher concentrations (70–90%) initially dissolved stones faster but degraded more rapidly, reducing total dissolution. No effect was seen in control (0%) stents. The stents gradually release agents, avoiding toxicity peaks while maintaining structural function; thus, maintaining biliary drainage, which facilitates CBD stone clearance.
**Buscemi (2017) **[16]	In vivo (D5; 10/20 points)	Biliary duct repair in rabbits using PHEA-PLA/PLC tubular scaffold.	PHEA-PLA/PCL conduits demonstrated notable resistance to bile’s corrosive action, while effectively supporting cellular infiltration and neovascularization. Additionally, these scaffolds facilitated epithelial cell stratification and sub-epithelial growth of accessory gland cells, thereby showing significant potential for regenerative biliary applications.
**Justin (2018) **[27]	Review (D5)	Bile duct bioengineering; assessment of various technologies.	This review highlights significant advancements in the field, emphasizing recent progress in tissue engineering and cholangiocyte culture. Key technological parameters evaluated regards cell types, materials, fabrication techniques and vascular supply. Moreover, it differentially assessed the results regarding acellular scaffolds and the ones seeded with BMCs and cholangiocytes, showing pros and cons of each approach.
**Huang (2019) **[28]	In vitro and in vivo (D5; 11/20 points)	Development of drug-eluting metal stent, coated with EDTA- and SC-loaded PCL, to promote biliary stone dissolution. Three different construction methods (1 = dip coating, 2 = coaxial electrospinning and 3 = both) were deployed and compared in vitro. In vivo, only stent biocompatibility is assessed, due to high mortality rates found in preliminary efficacy tests, when stones were inserted with stents to realize a stone-dissolution model.	Stents 1 and 2 showed a burst release of drugs in 5 days, while stent 3 presented controlled and sustainable drug release for 30 days. Stent 3 significantly leads to the most stone mass-loss, both in still buffer and in flowing bile models. In vivo, all animals survived after the stent placement. Hematological analyses were unalerted by the procedure. Histological analyses show physiological post-op. gallbladder and CBD inflammation, with normal duodenal wall, liver, and kidney.
**Girard (2020) **[29]	In vitro, in vivo and human cadaveric (D5)	Design of a resorbable biliary stent to reduce biliary complications after liver transplant. PLA/PEG copolymer was loaded with radiopaque triiodobenzoate(TIB)-PCL and tested in vitro, in vivo and on human cadavers.	In vivo biocompatibility test resulted optimally. In vivo and in vitro degradation tests resulted similarly, with a fast and quasi-linear trend during the first 10 weeks, before stabilization. Notably, the degradation process regarded almost only PLA/PEG structure, not the TIB-PCL radiopaque group. Mechanical tests proved acceptable properties for up to 4 weeks, thus, allowing bile to flow and CBD to heal before its degradation, even after X-ray irradiation. Accordingly, histological examinations up to 6 months showed a gradually degraded (and lately fragmented) stent, surrounded by a thin fibrotic tissue and with little leucocyte infiltrations, devoid of proper chronic inflammation process. Finally, 4 surgeons tested the implantability on cadavers, obtaining 75% success, confirmed by CT imaging.
**Brevini (2020) **[30]	Review (D5)	Tissue engineering of CBD.	PLA reported as synthetic material for CBD scaffolding, along with other polymers. Such materials might be fine-tuned, to successfully cope with the needs of regenerating bile ducts and cholangiocytes. Still, only a couple of pre-clinical applications are reported.
**Wang (2021) **[31]	Review (D5)	Tissue-engineered bile ducts (TEBDs) for disease modeling and therapy.	TEBDs are emerging as alternatives to conventional reconstruction. While various strategies aim to generate functional cholangiocytes (e.g., via stem cell technologies), scaffold technologies remain central to successful regeneration. Optimal scaffolds must be biocompatible, biodegradable, mechanically stable, and promote cell adhesion, proliferation, and differentiation. Natural materials offer biocompatibility but lack mechanical strength, whereas synthetic polymers such as PCL, PLA, PGA, and their copolymers provide tunable degradation and structure. Fabrication methods like electrospinning and 3D printing enable tailored architecture. Matching scaffold degradation to tissue remodeling is essential to avoid collapse. Hybrid scaffolds combining natural and synthetic elements show promise for balanced performance. Standardization and further preclinical studies are needed for clinical translation.
**Buisson (2022) **[32]	In vitro and in vivo (D5; 11/20 points)	Human chemically derived hepatic progenitor cells (hCdHs) were used to obtain induced cholangiocytes (hCdH-Chols); these cells were eventually seeded in a tubular, fine-tuned 3D-printed PCL scaffold. The latter presents an inner fibrous layer to enhance cell attachment, and a microporous outer layer to enhance mechanical properties. Such engineered CBD was tested both in vitro and on rabbit models.	In vitro, hCdH-Chols always proved successful differentiation and biliary functionality, while the scaffold supported cell adhesion, viability, and differentiation. Such fiber architecture enhanced biliary marker expression, reduced progenitor markers, and improved cell–matrix integration. Indeed, complete endothelization occurred in just 14 days. In 13 rabbit models, a CBD defect was created and further replaced with such scaffolds (5 seeded tests + 8 nude controls). This led to 80% survival at 42 days (1/5 tests died for post-op. complications), restoration of bile flow, and normalized liver function markers. Conversely, all controls died within 5 days. Histology and immunostaining confirmed graft integration, absence of strictures, and persistence of functional hCdH-Chols across the anastomosis site. The construct maintained shape and patency and allowed bile passage into the intestine.
**Hallett (2022) **[33]	In vitro and in vivo (D5; 17/20 points)	Development of a new cell line of human biliary epithelial cells (hBECs) from discarded cadaveric livers, to form induced cholangiocytes in vitro. Animal tests were conducted on knockout mouse models. hBECs were then tested on electrospun nanofibrous PCL scaffold.	In vitro, hBECs retained a stable cholangiocyte phenotype, high proliferative and clonogenic capacity, and bipotential features. In vivo, transplanted hBECs engrafted near bile ducts, reduced fibrosis, inflammation, and bilirubin levels, and improved survival compared to controls (PBS or hMSCs). Further in vitro tests confirmed the viability of such cells on PCL scaffold.
**Kim (2022) **[34]	In vivo (D5; 12/20 points)	PCL and barium sulfate biliary stent; 3D-printed and implanted in pigs.	Three pigs (one in the stent group and two in the control group) died within one day after surgery. Radiological follow-up of the 11 remaining pigs showed no evidence of bile duct obstruction or leakage, but computed tomography (CT) scans revealed three migrations and three fractures of the implanted stents. Moreover, histopathological analysis showed mild-to-moderate inflammation and increased fibrosis in stented animals compared to controls.
**Miyazawa (2022) **[35]	Review (D5)	Bile duct substitutes; assessment of various technologies.	In this review, key parameters evaluated for effectiveness include animal type, bile duct size, observation period, bile duct regeneration, and causes of stenosis. The bile duct substitutes are categorized based on the materials used: autologous tissue, non-bioabsorbable and bioabsorbable materials, natural or synthetic polymers, and other materials. For future scaffold development, critical considerations highlighted are bile duct wound healing, bile properties, bile duct and anastomotic site regeneration. These factors are essential for ensuring both short-term functionality and long-term safety and efficacy of the substitutes.
**De Siervi (2023) **[36]	Review (D5)	In vitro model to study primary liver cancer.	Scaffold-based 3D systems provide a biomimetic microenvironment that promotes tumor cell aggregation, proliferation, and migration by mimicking ECM architecture. Natural scaffolds (e.g., Matrigel, hyaluronic acid, collagen, gelatin) offer biocompatibility, whereas synthetic polymers (e.g., PEG, PLA, PCL and others) ensure tunable mechanical and chemical properties, reproducibility, and controlled degradation. These features make synthetic scaffolds particularly suitable for modeling tumor behavior and testing anti-cancer agents under standardized conditions, with reduced batch variability and enhanced experimental reliability compared to natural matrices.
**Valderrama-Treviño (2024) **[37]	In vivo (D5)	Extrahepatic bile duct reconstruction; PLGA–PCL–Gel scaffold implanted in pigs.	This study proved successful scaffold integration over an 18-month period. Previous mechanical and biological tests confirmed in vitro the potential of the device. Histological analyses confirmed the presence of biliary epithelium, indicating effective regeneration and integration of the scaffold into the bile duct system.

**Table 2:** Description of study groups.

Group	Scaffold Type	Nr. of Animals	Follow-Up	Endpoint
**A**	Planar, gallbladder	5	3 months	Programmed euthanasia
**B**	Tubular, gallbladder-jejunal	5	3 months	Programmed euthanasia
**C**	Tubular, CBD	5	3 months	Programmed euthanasia
**D**	Patch, CBD	5	1 month	Programmed euthanasia

**Table 3:** Semiquantitative histopathological results. Here, the number of “+” symbols represents the intensity of response obtained. These are shown with a color code, with green representing an optimal response, yellow an intermediate response, and red a suboptimal response.

Groups	Inflammatory Infiltrate	Neovascularization	Epithelial Regeneration	Scaffold Degradation
**A—Planar, gallbladder**	**++**	**+++**	**+++**	**++**
**B—Tubular, gallbladder-jejunal**	**+++**	**++**	**++**	**+**
**C—Tubular, CBD**	**++**	**++**	**++**	**++**
**D—Patch, CBD**	**+**	**+++**	**+++**	**+**

**Table 4:** Cochrane Library Search String.

ID	Search	Hits
**#1**	Bile Ducts	816
**#2**	Biliary Tract	2587
**#3**	Common Bile Duct	1688
**#4**	CBD	1825
**#5**	CBD injuries	34
**#6**	CBD repair	14
**#7**	Biliary injury repair	23
**#8**	Bile duct regeneration	7
**#9**	Biliary reconstruction	171
**#10**	Polymeric scaffold	24
**#11**	Bioabsorbable polymer	125
**#12**	Biodegradable scaffold	46
**#13**	Polycaprolactone	48
**#14**	PCL	1006
**#15**	Polyhydroxyethyl aspartamide	0
**#16**	PHEA	3
**#17**	Polylactic Acid	265
**#18**	PLA	4025
**#19**	Polylactide	98
**#20**	Tissue Engineering	1991
**#21**	Regenerative Medicine	760
**#22**	#1 OR #2 OR #3 OR #4 OR #5 OR #6 OR #7 OR #8 OR #9	5986
**#23**	#10 OR #11 OR #12 OR #13 OR #14 OR #15 OR #16 OR #17 OR #18 OR #19 OR #20 OR #21	8097
**#24**	#22 AND #23	36

## Data Availability

The original contributions presented in the study are included in the article/Supplementary Materials, further inquiries can be directed to the corresponding author.

## Supplementary Materials

The following supporting information can be downloaded at: https://www.mdpi.com/article/10.3390/jfb16080297/s1, Table S1: PRISMA 2020 Checklist; Table S2: ARRIVE Checklist.
